# Pediatric AML: From Biology to Clinical Management

**DOI:** 10.3390/jcm4010127

**Published:** 2015-01-09

**Authors:** Jasmijn D. E. de Rooij, C. Michel Zwaan, Marry van den Heuvel-Eibrink

**Affiliations:** Department of Pediatric Oncology, Erasmus MC-Sophia Children’s Hospital, 3015CN Rotterdam, The Netherlands; E-Mails: j.d.e.derooij@erasmusmc.nl (J.D.E.R.); c.m.zwaan@erasmusmc.nl (C.M.Z.)

**Keywords:** pediatric AML, clinical management, cytogenetics, molecular aberrations

## Abstract

Pediatric acute myeloid leukemia (AML) represents 15%–20% of all pediatric acute leukemias. Survival rates have increased over the past few decades to ~70%, due to improved supportive care, optimized risk stratification and intensified chemotherapy. In most children, AML presents as a *de novo* entity, but in a minority, it is a secondary malignancy. The diagnostic classification of pediatric AML includes a combination of morphology, cytochemistry, immunophenotyping and molecular genetics. Outcome is mainly dependent on the initial response to treatment and molecular and cytogenetic aberrations. Treatment consists of a combination of intensive anthracycline- and cytarabine-containing chemotherapy and stem cell transplantation in selected genetic high-risk cases or slow responders. In general, ~30% of all pediatric AML patients will suffer from relapse, whereas 5%–10% of the patients will die due to disease complications or the side-effects of the treatment. Targeted therapy may enhance anti-leukemic efficacy and minimize treatment-related morbidity and mortality, but requires detailed knowledge of the genetic abnormalities and aberrant pathways involved in leukemogenesis. These efforts towards future personalized therapy in a rare disease, such as pediatric AML, require intensive international collaboration in order to enhance the survival rates of pediatric AML, while aiming to reduce long-term toxicity.

## 1. Clinical Introduction

### 1.1. Epidemiology of AML

In children, the most frequently occurring hematological malignancies include acute leukemias, of which 80% are classified as acute lymphoblastic leukemia (ALL) and 15%–20% as acute myeloid leukemia (AML). The incidence of AML in infants is 1.5 per 100,000 individuals per year, the incidence decreases to 0.9 per 100,000 individuals aged 1–4 and 0.4 per 100,000 individuals aged 5–9 years, after which it gradually increases into adulthood, up to an incidence of 16.2 per 100,000 individuals aged over 65 years [1]. The underlying cause of AML is unknown, and childhood AML generally occurs *de novo*. In adult and elderly patients, AML is often preceded by myelodysplastic syndrome (MDS), but in children, the occurrence of AML preceded by clonal evolution of preleukemic myeloproliferative diseases, such as MDS or juvenile myelomonocytic leukemia (JMML), is rare. Germline affected individuals, such as those with Fanconi anemia or Bloom syndrome, have an increased risk for developing AML as a secondary malignancy [2,3]. Recently, germ-line mutations in several genes, such as TP53, RUNX1, GATA2 and CEBPA, have been found in families with an unexplained high risk of AML, suggesting a familial predisposition to develop AML [4,5,6,7,8].

Children with Down syndrome classically present with a unique megakaryoblastic subtype of AML, classically following a transient myeloproliferative disorder in the neonatal period, which is characterized by somatic mutations in the *GATA1* gene. The leukemic cells of patients with Down syndrome are usually highly sensitive to chemotherapy with an exceptional high survival rate, and therefore it is possible to treat these patients with adjusted treatment protocols [9]. In addition, AML may occur following previous radiotherapy or chemotherapy containing alkylating agents or epipodophyllotoxins, as secondary neoplasm. These are typically characterized by either MLL-rearrangements or by monosomy 7 [10,11].

### 1.2. Diagnostic Approach and Classification

AML is a heterogeneous disease with respect to morphology, immunophenotyping, cooperating underlying germline and somatic genetic abnormalities, as well as clinical behavior. The standard diagnostic process of AML is based on a combination of morphology, cytochemistry, immunophenotyping, cytogenetic and molecular characterization of the leukemic blasts derived from the bone marrow or peripheral blood [12]. Each AML patient can be risk-classified into a clinically relevant subgroup. The previously used morphology-based French-American-British (FAB) classification is nowadays replaced by the World Health Organization (WHO) classification, which also takes karyotype and molecular aberrations into account (Table 1) [13,14]. Cytochemistry and immunophenotyping is generally used to distinguish AML from ALL, which further classifies pediatric AML according to the cell lineage of origin and differentiation stage at which the differentiation arrest occurs. Especially for the diagnosis of FAB-types, M0 and M7 immunophenotyping is indispensable [12,15]. The majority of chromosomal abnormalities is detected by conventional karyotyping and complemented with FISH or reverse transcriptase PCR to detect relevant (cryptic) translocations, fusion genes or loss of chromosome material [16]. In young children under two years of age, it is important to search for specific pediatric AML translocations that are not yet acknowledged in the WHO classification as separate entities, such as t(7;12)(q36;p13), also known as *HLXB9-MNX1*, t(11;12)(p15;p13)/*NUP98-KDM5A* and t(1;22)(p13;q13)/*RBM15-MKL1* [12,17,18,19].

**Table 1:** The WHO classification of acute myeloid leukemia (AML) and related neoplasms [14].

WHO Classification of AML and Related Neoplasms	
Acute myeloid leukemia with recurrent genetic abnormalities	AML with t(8;21)(q22;q22); *RUNX1-RUNX1T1*
	AML with inv(16)(p13.1q22) or t(16;16)(p13.1;q22); *CBFB-MYH11*
	Acute promyelocytic leukemia with t(15;17)(q22;q12); *PML-RARA*
	AML with 11q23 (*MLL)* abnormalities
	AML with t(6;9)(p23;q34); *DEK-NUP214*
	AML with inv(3)(q21q26.2) or t(3;3)(q21;q26.2); *RPN1-EVI1*
	t(1;22)(p13;q13); *RBM15-MKL1*
	Provisional entity: AML with mutated *NPM1*
	Provisional entity: AML with mutated *CEBPA*
Acute myeloid leukemia with myelodysplasia-related changes	
Therapy-related myeloid neoplasms	
Acute myeloid leukemia, not otherwise specified	AML with minimal differentiation
	AML without maturation
	AML with maturation
	Acute myelomonocytic leukemia
	Acute monoblastic/monocytic leukemia
	Acute erythroid leukemia
	Pure erythroid leukemia
	Erythroleukemia, erythroid/myeloid
	Acute megakaryoblastic leukemia
	Acute basophilic leukemia
	Acute panmyelosis with myelofibrosis
Myeloid sarcoma	
Myeloid proliferations related to Down syndrome	Transient abnormal myelopoiesis
	Myeloid leukemia associated with Down syndrome
Blastic plasmacytoid dendritic cell neoplasm	

### 1.3. Treatment and Outcome

The clinical outcome of pediatric AML has improved significantly over the past few decades, with current long-term survival rates of ~70% (Table 2) [20,21,22,23,24,25,26,27,28,29,30]. This improvement is due to intensification of chemotherapeutic regimens, better risk-group stratification, better salvage at relapse and improved supportive care. Risk-group stratification is usually based on (cyto)genetic abnormalities present in the leukemic blasts in combination with early response to treatment, either specified as complete remission (CR) rate after one or two courses or applying minimal-residual disease measurements, which in AML is mainly based on flow-cytometry [31]. The chemotherapeutic regimens consist of 4–5 cycles of intensive chemotherapy, typically including cytarabine combined with an anthracycline. In younger adult patients, studies suggest that there is a benefit for outcome using high-dose cytarabine in induction, but from previous trials, a similar effect in pediatric AML patients could not be confirmed [26,32]. In randomized controlled trials, the anthracyclines, daunorubicin and mitoxantrone, resulted in similar overall survival, but mitoxantrone-based treatment eventually resulted in a lower relapse rate [33]. When comparing idarubicin and liposomal daunorubicin, survival was similar, whereas liposomal daunorubicin was more effective in *RUNX1/RUNX1T1* translocated cases and caused less treatment-related mortality [34].

The added value of hematopoietic stem cell transplantation (SCT) in newly-diagnosed pediatric AML is under discussion, as in general, the occurrence of procedure-related deaths needs to be counterbalanced by the reduction in relapse risk. The procedure-related deaths are dependent on the intensity of the prior induction chemotherapy. SCT in first CR is therefore currently only recommended for a selected subset of high risk cases in most European protocols. SCT plays a more prominent role in most North-American treatment protocols [35,36]. Recent studies show an increase in survival after SCT now that stricter risk stratification is improving. Currently, several trials include minimal residual disease (MRD) levels after Courses 1 or 2 in risk stratification for SCT [37,38,39,40]. Of note is that the excess in mortality and the burden of disease long after myeloablative therapy are not taken into account in the reported survival.

Despite intensive treatment, ~30% of the pediatric patients relapse, and outcome is poor, reflected by the ~30%–40% of patients surviving in the largest and most recent series reported to date [41,42]. Nevertheless, the high frequency of treatment-related deaths (5%–10%), both in treatment protocols for newly-diagnosed, as well as for relapsed disease, and the occurrence of long-term side effects, such as anthracycline-induced cardiomyopathy, illustrate that further intensification of chemotherapy seems no longer feasible [43]. Therefore, knowledge on the molecular and genetic background is of utmost relevance in order to detect novel, leukemia and patient-specific treatment targets.

### 1.4. Relevant Molecular and Genetic Aberrations in Pediatric AML

**Figure 1:**
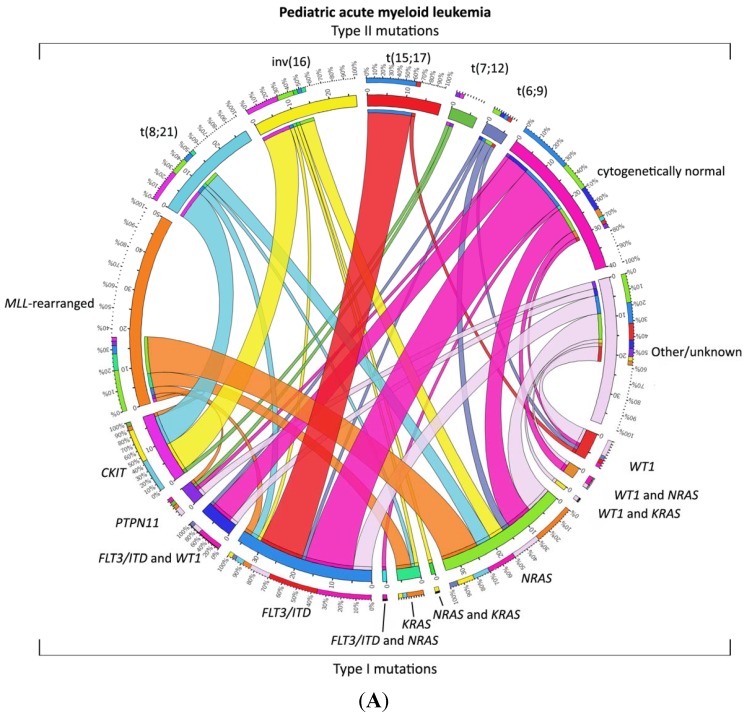

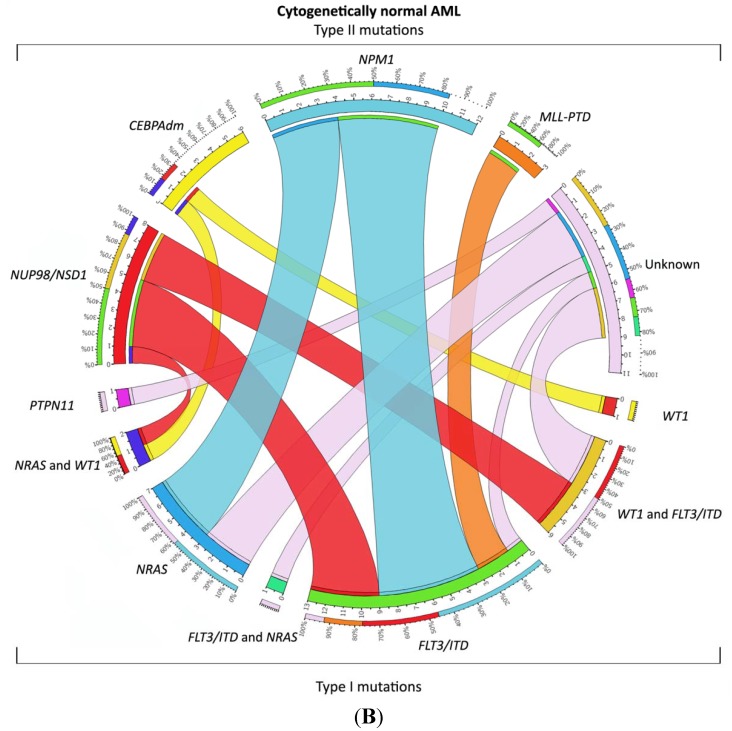
Distribution of Type I/II abnormalities in pediatric AML. (**A**) Cooperating Type I and Type II mutations in pediatric AML. The circos plot [73] depicts the frequency of the Type II mutations and co-occurrence of Type I mutations in patients with *de novo* pediatric AML. The length of the arch corresponds to the frequency of the Type II mutation and the width of the ribbon with the percentage of patients with a specific Type I mutation or a combination of Type I mutations. FLT3/ITD denotes FLT3 internal tandem duplication; (**B**) Cooperating Type I and Type II mutations in cytogenetically normal AML. The circos plot [73] depicts the frequency of the Type II mutations and co-occurrence of Type I mutations in patients with *de novo* pediatric cytogenetically normal AML. The length of the arch corresponds to the frequency of the Type II mutation, and the width of the ribbon with the percentage of patients with a specific Type I mutation or a combination of Type I mutations. FLT3/ITD denotes FLT3 internal tandem duplication.

AML is thought to arise from at least two classes of cooperating genetic events [44]. Type I abnormalities result in increased, uncontrolled proliferation and/or survival of the leukemic cell and are often activating mutations of genes involved in signal transduction pathways, such as *FLT3*, *KIT*, *N-RAS*, *K-RAS* and *PTPN11*. Type II abnormalities impair differentiation and mainly result from genetic aberrations in hematopoietic transcription factors, due to, for instance, the AML-characteristic translocations t(8;21)(q22;q22)/*AML1-ETO* and 11q23/*MLL* rearrangements or from mutations in genes, such as *NPM1* and *CEBPA* [7,45,46,47,48]. The most common cytogenetic abnormalities (Type II) in children are t(8;21)(q22;q22), inv(16)(p13.1q22) (together referred to as core binding factor (CBF)-AML), t(15;17)(q22;q21) and 11q23/*MLL*-rearranged abnormalities (Figure 1A) [49,50,51,52]. Together, these account for approximately half of all pediatric AML cases, a much higher frequency than in adults. Some translocations, for example t(1;22)(p13;q13), t(7;12)(q36;p13) and t(11;12)(p15;p13), are specific for children and are rarely or never found in adults [17,18,19,53,54,55,56,57]. Translocations involving hematopoietic transcription factors often lead to dysregulated gene expression, either as a result of the fusion partner itself or the recruitment of different co-factors to the transcription complex. For example, the *MLL* gene has histone methyltransferase activity and is part of a chromatin modifying complex. More than 60 fusion partners have been identified in AML, but the breakpoint of the *MLL* gene is highly conserved [58,59]. Fusion proteins lead to a gain of function of the *MLL*-complex, resulting in inappropriate histone modification and increased expression of *MEIS1* and, specifically, *HOXA* genes, maintaining a stem-cell phenotype. In addition, the presence of DOT1L, which is recruited into the *MLL-*complex, is required for the leukemogenic activity of several *MLL* rearrangements and may be a target for treatment [60,61].

Only 20%–25% of pediatric AML cases are cytogenetically normal [48,62]. Of interest in these cases, specific Type II mutations and translocations are identified in ~70% of the cases, such as *NPM1* mutations, biallelic *CEBPA* mutations, as well as the cryptic translocations, *NUP98/NSD1*, all invisible with conventional karyotyping and, hence, requiring additional molecular diagnostics (Figure 1B) [17,46,57,63].

The combination of the Type I and Type II mutations does not seem to be completely random; specific combinations seem more prevalent, such as the Ras pathway mutations, which are often found in combination with *MLL-*rearrangements, *KIT* mutations, which are mainly found in CBF-AML, and *FLT3-*itd, which is often seen in combination with *PML/RARA* and *NUP98/NSD1* (Figure 1) [48,57].

**Figure 2:**
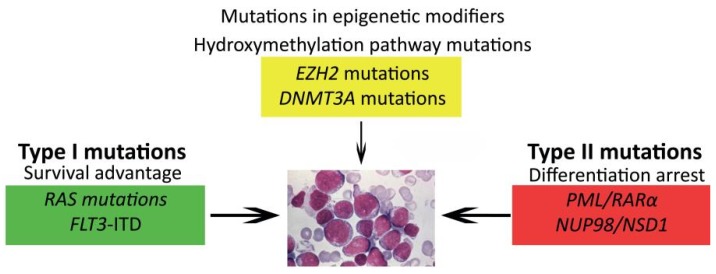
Model of cooperating genetic events in AML. Different types of genetic and epigenetic events collaborate in leukemogenesis.

Mutations in epigenetic regulators, such as *EZH2*, *ASXL1* and *DNMT3A*, add another level of complexity and contribute to both the maturation arrest and proliferative capacity, which are needed to develop AML (Figure 2) [63,64,65,66,67,68,69,70,71]. These mutations are rare in pediatric AML, but specific Type II subgroups present with an altered methylation (hypo- or hyper-methylation), which may indicate that these children could benefit from treatment with demethylating agents or histone modification inhibitors, as recently described for infants suffering from ALL [72].

**Table 2:** Survival of pediatric AML.

Study Group	Study and Inclusion Time (Calendar Years of Inclusion)	Patients (*n*)	Patients Treated with SCT (*n*)	EFS (%)	OS (%)	Relapse (%)	Source
BFM-SG	AML-BFM 2004 (2004–2010)	521	NA	5 years 55 ± 2	5 years 74 ± 2	29	Creutzig *et al.*, 2013 [34]
JACLS	AML99 (2003–2006)	146	22 (15%)	5 years 66.7 ± 4.0	5 years 77.7 ± 8.0	30.2	Imamura *et al.*, 2012 [74]
	AML99 (2000–2002)	240	Allo-SCT 41 (17%) Auto-SCT 5 (2%)	5 years 61.6 ± 6.5	5 years 75.6 ± 5.3	32.2	Tsukimoto *et al.*, 2009 [27]
AIEOP	AML2002/01 (2002–2011)	482	Allo-SCT 141 (29%) Auto-SCT 102 (21%)	8 years 55.0 ± 2.6	8 years 67.7 ± 2.4	24	Pession *et al.*, 2013 [30]
COG	AAML03P1 (2003–2005)	340	73 (21%)	3 years 53 ± 6	3 years 66 ± 5	33 ± 6	Cooper *et al.*, 2012 [75]
NOPHO	NOPHO AML 2004 (2004–2009)	151	22 (15%)	3 years 57 ± 5	3 years 69 ± 5	30	Abrahamsson *et al.*, 2011 [20]
MRC	MRC AML12 (1995–2002)	564	64 (11%)	10 years 54	10 years 63	32	Gibson *et al.*, 2011 [33]
SJCRH	AML02 (2002–2008)	216	59 (25%)	3 years 63	3 years 71	21	Rubnitz *et al.*, 2010 [26]
PPLLSG	PPLLSG AML-98 (1998–2002)	104	Allo-SCT 14 (13%) Auto-SCT 8 (8%)	5 years 47 ± 5	5 years 50 ± 5	24	Dluzniewska *et al.*, 2005 [76]

Abbreviations: *n*, indicates number; SCT, stem cell transplantation; EFS, event-free survival; OS, overall survival; BFM-SG, Berlin-Frankfurt-Munster-Study-Group (Germany); AML, acute myeloid leukemia; JACLS, Japan Association of Childhood Leukemia Study; Allo, allogeneic; Auto, autologous; AIEOP, Italian association of Pediatric Hematology and Oncology (Associazione Italiana Ematologia Oncologia Pediatrica); COG, Childhood Oncology Group (United States of America); NOPHO, Nordic Society of Pediatric Haematology and Oncology; MRC, Medical Research Council (United Kingdom); SJCRH, St. Jude Children’s Research Hospital (United States of America); PPLLSG, Polish Pediatric Leukemia/Lymphoma Study Group.

### 1.5. Prognostic Factors and Risk Group Stratification

Most important prognostic factors for the survival of pediatric AML are the initial response to treatment and the underlying genetic and molecular aberrations [12,77,78]. CBF-AML is a favorable prognostic subgroup [48,52,79]. Outcome in *MLL*-rearranged AML is variable and depends on the translocation partner. For example, the *MLL*-translocation t(1;11)(q21;q23) is associated with a very favorable outcome in pediatric AML. In contrast, poor survival rates were reported in pediatric AML with translocations t(6;11)(q27;q23) and t(10;11)(p12;q23) [80,81]. The acute megakaryoblastic leukemias (AMKL, FABM7) in non-Down syndrome patients represent a subgroup with poor outcome, with the exception of AMKL harboring t(1;22)(p13;q13), which seems to confer a favorable prognostic group, in contrast to Down syndrome, where AMKL confers a favorable outcome [9,17]. Monosomy 7 is a well-known poor-prognostic factor and confers a worse outcome [52,82]. Deletion of 7q is described as an intermediate risk in the prognosis in adults, in contrast to the outcome of pediatric AML with a 7q deletion in children. In those pediatric patients, the outcome seems to be dependent on other cytogenetic abnormalities in the leukemic cell [52,82]. The described poor prognostic abnormalities in adult AML of chromosomes 3q and 5q and the monosomal karyotypes are rare in children [30,83,84,85]. Overexpression of *EVI1* caused by 3q26 abnormalities predicts an adverse outcome in adult AML, but *EVI1* overexpression is not an independent prognostic factor in pediatric AML [86,87]. The Type I mutations of *WT1* and *FLT3-*itd predict a poor outcome, the latter dependent on the allele ratio, and these mutations are described as events in clonal evolution towards relapse [88].

A special subtype of pediatric AML is the cytogenetically normal (CN) AML group, where clinical outcome is highly dependent on the presence of single-gene mutations or cryptic translocations. Of special interest are *NPM1* and bi-allelic *CEBPA* mutations, conferring a favorable prognosis, while the cryptic translocation *NUP98/NSD1* confers a poor prognosis, due to a poor response to treatment and a high risk for relapse, independent of the poor prognostic Type I *FLT3-*itd abnormality [46,57,89].

## 2. Future Strategies

### 2.1. Genomic Approaches to Unravel the Biology of Pediatric AML

In order to provide more insight into the heterogeneity and biology of AML, genome-wide approaches have been recently employed, although the success rate is variable. Array-based comparative genomic hybridization (array-CGH) and single-nucleotide polymorphism (SNP) arrays identified several regions with loss of heterozygosity and recurrent copy number variations (CNVs), albeit with low frequency in AML [90]. These CNVs included aberrations in *WT1*, *NF1* and *TET2*, the latter being more common in adults than in children [47,65,67,91].

Gene expression profiling could predict the cytogenetic subtypes of AML with high accuracy, although its value for diagnostic purposes remains limited, since most aberrations can be identified with conventional karyotyping [92,93,94,95]. Nevertheless, novel genes involved in the pathogenesis of pediatric AML subtypes were identified using this method, such as *BRE* and *IGSF4* [96,97].

In addition to discovering novel gene mutations, next generation sequencing has also proven to be a powerful tool in the study of the clonal evolution of both adult and pediatric AML [98,99]. By comparing the mutational spectrum of diagnosis-relapse pairs, it was shown that the founding clone gained novel mutations and evolved into the relapse clone. Moreover, minor subclones present at diagnosis can survive chemotherapy, gain mutations and present as dominant clones at relapse, illustrating their leukemia-driving capacity. Therapeutic targeting of novel identified mutations to prevent relapse may provide an improved outcome for selected patients [100,101].

Epigenetic profiling was able to distinguish cytogenetic subtypes of adult AML [102]. Differences in promoter hypermethylation of selected genes between pediatric and adult AML warrant the profiling of DNA methylation in pediatric AML [103]. These studies may point out subsets of patients eligible for treatment with demethylating agents or histone modification inhibitors, as was shown for pediatric ALL [72].

Differences in microRNA expression levels can classify several types of cancer [104]. Profiling studies in adult AML have shown that variations in microRNA expression patterns are associated with subtypes of AML and that specific microRNAs target genes of interest for the biology of AML [105,106,107]. In pediatric AML, microRNA expression patterns vary among subtypes of AML, as well, although some differences in the expression patterns of specific microRNAs were observed between children and adults [108].

### 2.2. Towards Optimized Therapy

The translation from molecular aberrations towards targeted therapy might be the solution to improve outcome in the next few decades. Since further intensification of current chemotherapy treatment seems not feasible in pediatric AML, due to high morbidity and mortality rates, new therapeutic approaches that are more tumor-specific and cause less severe side effects are urgently needed. Some new compounds directed at specific molecular targets have already been investigated in early clinical trials in pediatric AML.

Tyrosine kinase inhibitors directed at inhibiting the constitutive activation of the *FLT3* gene are among the best studied drugs in this respect in pediatric AML and include trials using PKC412, CEP701, AC220 and sorafenib [109,110,111]. Recent data suggest a potentially generic mechanism of drug resistance when combining these inhibitors with chemotherapy due to FLT3 ligand upregulation, which questions their use in this fashion, although novel, more potent inhibitors may overcome this [112,113]. In the AAML1031 study of the Childhood Oncology Group (COG), patients with a *FLT3* gene mutation are treated with sorafenib in addition to standard intensive chemotherapy [114]. However, there are no convincing randomized studies to date showing an increase in overall survival in *FLT3* mutated patients with such therapeutic regimens.

Other potential targets in AML include *KIT* and *RAS* gene mutations. Patients with *KIT* mutations include the imatinib-resistant patients with the *D816V/Y* mutation, who are sensitive to dasatinib [48,115]. A phase I study of dasatinib has been completed in children [116]. There is an ongoing trial in adults using dasatinib together with chemotherapy in CBF-AML [117]. No trials have been reported using small molecule RAS-pathway inhibitors, e.g., MEK-inhibitors, after studies using farnesyl transferase inhibitors failed to show a benefit in older patients with AML [118]. To inhibit signal transduction pathways, such as the Ras-pathway, which is notorious for escaping behavior, which makes the leukemic cell survives despite intensive chemotherapy, combinations of inhibitors may be more promising, and this approach is currently being further explored in synthetic lethality screens, which combines different inhibitors in order to find a lethal combination for the leukemic cells, for different types of cancer [119,120].

In *MLL-*rearranged AML, efforts are directed at developing targeted therapy, for instance by inhibiting DOT1L, which is part of the MLL-complex, with current clinical trials ongoing [60]. Interestingly, these DOT1L inhibitors also seem valuable in the treatment of t(6;11)(q27;q23)-positive cells, which lack DOT1L in the formed complex, indicating that this drug is able to target aberrant H3K79 methylation [121].

Gemtuzumab ozogamicin is a conjugated monoclonal antibody against CD33 and linked to a cytostatic agent, calicheamicin, an anti-tumor antibiotic. AML cells often express CD33 and are therefore targeted by this drug. The first phase III studies did not show an improvement in disease-free and overall survival in pediatric AML patients, but it was favorable in patients with refractory or relapsed disease and was effective at reducing MRD levels before SCT [75,122,123,124,125].

Clofarabine is a purine nucleoside antimetabolite, registered for relapsed or refractory pediatric ALL. Early trials in pediatric AML did not show convincing efficacy, probably due to the intensive pre-treatment in these cases [126]. However, in refractory or relapsed pediatric AML patients, the combination of clofarabine and cytarabine resulted in 48% responders, with a three-year overall survival of 46% [127]. In ongoing studies, fludarabine, used in the “FLAG”-therapy, consisting of fludarabine, cytarabine and G-CSF, is replaced by clofarabine, as well as clofarabine combined with cyclophosphamide and etoposide [128]. Another phase II study showed a beneficial outcome for patients treated with the combination of clofarabine, topotecan, vinorelbine and thiotepa in pediatric patients with refractory or relapsed AML [129].

In xenograft models, treatment of AML with a combination of decitabine, a hypomethylating agent, and cytarabine was more effective at reducing tumor burden in comparison to cytarabine alone [130]. Low-dose decitabine was administered to high-risk relapsed or refractory AML patients, and 3/8 patients responded to this therapy [131]. Azacitidine, another hypomethylating agent, and decitabine show comparable treatment efficacy, but azacitidine may result in less adverse events [132].

International collaboration, which has been pursued over the last few decades on the levels of the International Berlin-Frankfurt-Munster Study Group (IBFM-SG), Innovative Therapies for Children with Cancer (ITCC), European Network for Cancer Research in Children and Adolescents (ENCCA), Therapeutic Advances in Childhood Leukemia (TACL) and Childhood Oncology Group (COG), has been proven successful in clinical and biological studies and will speed up efforts to enhance therapeutic options and the availability of novel agents for individual pediatric AML patients [12,17,28,80,81,82].

## 3. Conclusions

Current survival of pediatric AML is ~70%, and a therapeutic plateau has been reached with current chemotherapy. Further intensification of treatment is not feasible because of toxicity. The heterogeneity of AML is illustrated by the various prognostically relevant non-randomly associated molecular and cytogenetic aberrations that were discovered in recent years. However, many cooperating events in leukemogenesis still remain unknown. The application of new techniques, especially next generation sequencing, will contribute to our understanding of the genetic landscape of AML and enable the development of more targeted and personalized therapy in the near future. To achieve such goals for such a rare disease as pediatric AML, international collaboration is crucial.
